# Supplementary material for: The Association between Postpartum Practice and Chinese Postpartum Depression: Identification of a Postpartum Depression-Related Dietary Pattern
Source: Nutrients. 2022 Feb 21;14(4):903. doi: 10.3390/nu14040903 (PMC8880681; doi:10.3390/nu14040903)
Supplement: Supplementary file 1 [file nutrients-14-00903-s001.zip › nutrients-1550526-supplementary.pdf]

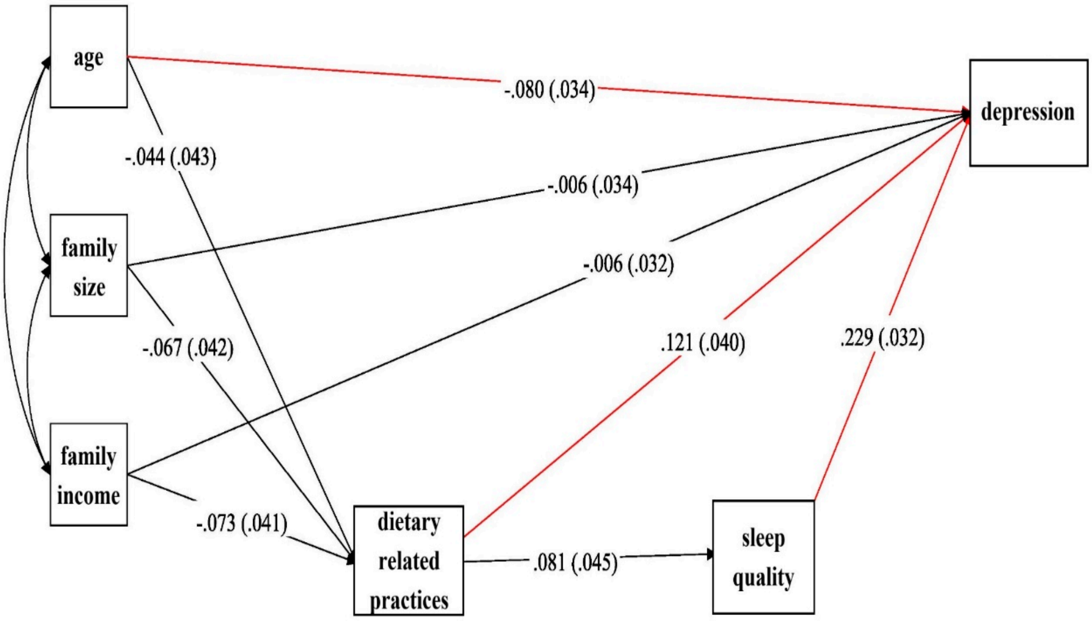

Supplementary Figure S1. The pathways among postpartum dietary practices and postpartum depression. The standardized effects (P values) are presented, and the red lines indicate the associations with significance.
